# Enablers, Challenges, and Lessons Learned From a Digital Health Intervention (Sehatmandi App) in Afghanistan: Qualitative Study

**DOI:** 10.2196/74923

**Published:** 2025-11-07

**Authors:** Saleem Sayani, Farah Jabeen, Saira Samnani, Abdul Muqeet, Amna Khan, Ghulamuddin Delawar, Meraj Subzlani

**Affiliations:** 1 Aga Khan University's Digital Health Resource Centre Aga Khan University Karachi, Sindh Pakistan; 2 Radiology Aga Khan University Karachi Pakistan; 3 International Rescue Committee Islamabad Pakistan; 4 Aga Khan Development Network Kabul Afghanistan

**Keywords:** mHealth, digital health evaluation, health system strengthening, sustainability, low- and middle-income countries, Afghanistan, qualitative research

## Abstract

**Background:**

In low- and middle-income countries, maternal, newborn, and child health face significant challenges due to infrastructure limitations, access disparities, and service delivery inefficiencies. The Sehatmandi mobile health (mHealth) app was deployed in 2018 to address these issues across 189 health facilities in Afghanistan's Bamyan and Badakhshan provinces. This app aims to enhance service provision through real-time data monitoring, improved accountability, and performance-based health system strengthening.

**Objective:**

This study aims to explore the enablers, challenges, and lessons learned for the sustainability of the Sehatmandi mHealth intervention from the perspective of key stakeholders to inform the future scaling of digital health tools in fragile and resource-constrained settings.

**Methods:**

A qualitative study was conducted between June and July 2024 involving 24 in-depth interviews with stakeholders, including health facility managers, administrators, and high-level decision-makers. Participants were selected using stratified purposive sampling to ensure diverse facility representation. Interviews were conducted in person or virtually by using a semistructured guide, recorded, transcribed and translated into English. Thematic content analysis was performed using NVivo version 11 software. Ethics approval was obtained, and informed consent was secured from all the participants.

**Results:**

Stakeholders reported that Sehatmandi improved health system responsiveness by enhancing performance monitoring, accountability, timely reporting, and data-driven decision-making. Offline data entry was identified as a critical feature, enabling data collection in remote areas without internet access and ensuring synchronization when connectivity was resumed. However, several barriers affected the long-term sustainability: poor internet connectivity, electricity shortages,inadequate technical support, and high staff turnover, which disrupted functionality and data quality. Training gaps and insufficient supervision further hampered consistent and effective use. Participants emphasized the need for structured capacity building, regular follow-up, and sustainable funding to maintain the intervention. Integration with national health information systems and alignment with broader digital health strategies were also seen as prerequisites for scaling and institutionalization.

**Conclusions:**

The Sehatmandi mHealth intervention demonstrated enhanced performance monitoring and accountability across health care facilities in Afghanistan’s conflict-affected settings. However, for digital health interventions to be sustainable and scalable in low- and middle-income countries, foundational investments in digital infrastructure, continuous training and monitoring, system-level integration, and long-term funding are essential. These findings provide actionable insights for governments, implementers, and donors aiming to strengthen health systems through digital innovation in fragile settings.

## Introduction

Maternal, newborn, and child health remains a global priority, particularly in low- and middle-income countries (LMICs), where preventable maternal and child mortality rates are high [1]. Despite progress in recent decades, LMICs face persistent barriers, including insufficient health care infrastructure, inequitable access to services, and inefficiencies in service delivery [2]. Mobile health (mHealth) interventions have emerged as transformative tools to address these challenges by improving health care accessibility, enhancing system efficiency and supporting data-driven decision-making [3]. These technologies have demonstrated their potential to empower health care workers, improve service delivery systems, and support evidence-based decision-making through real-time data monitoring [4]. However, the sustainability of mHealth solutions in resource-constrained settings is often hindered by inadequate digital infrastructure, limited digital literacy, insufficient integration with health systems, and funding constraints [5].

Afghanistan exemplifies these challenges, with some of the highest maternal, newborn, and child health mortality rates globally: maternal mortality at 638 per 100,000 live births, under-five mortality at 57.7 per 1000 live births, and infant mortality at 45 per 1000 live births [6]. The health system is critically short of qualified health care workers, which limits access to essential services for Afghan women, especially in conflict-affected areas [7]. To address this, the Ministry of Health of Afghanistan introduced the basic package health service and essential package hospital service [8,9]. The introduction of these programs led to the establishment of multiple health facilities and improved service utilization, including institutional deliveries and antenatal care coverage [10]. In 2018, with the support of donors, the Afghan Ministry of Public Health launched the Sehatmandi project, which means well-being in Dari. The goal was to improve the use and quality of health, nutrition, and family planning services across the country. As part of this project, health services were contracted out to nongovernmental organizations, and a pay-for-performance model was adopted. Under this model, service providers received financial compensation based on their performance in achieving targets for specific 11 health indicators, which improved the quality and monitoring of health care provision [8].

**Figure 1:**
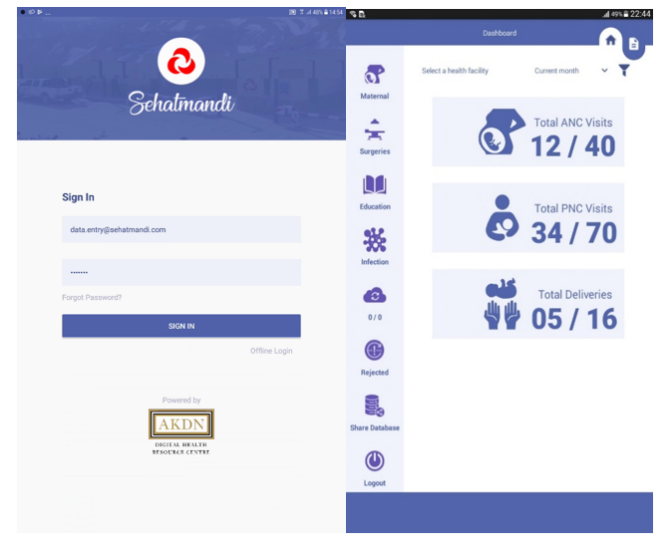
User Interface of the Sehatmandi mobile health app. ANC: antenatal care; PNC: postnatal care.

**Figure 2:**
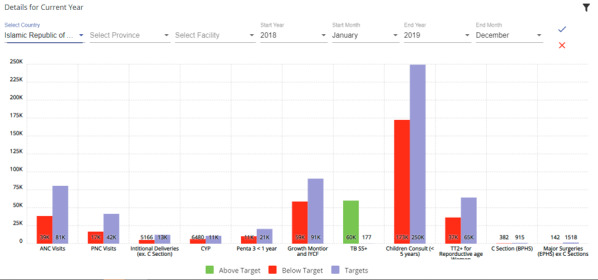
Dashboard view of the Sehatmandi application displaying key performance indicators for 2018–2019. The bar chart compares performance (above or below target) with predefined targets across health indicators. ANC: antenatal care; BPHS: Basic Package of Health Services; C-section: caesarean section; CYP: couple-years of protection; EPHS: Essential Package of Hospital Services; IYCF: infant and young child feeding; Penta 3: third dose of the pentavalent vaccine; PNC: postnatal care; TB SS+: sputum-smear positive tuberculosis; TT2+: tetanus toxoid, two or more doses.

As part of this broader initiative, in 2019, Aga Khan Health Services, Afghanistan, an NGO, was entrusted with the responsibility of managing 189 government health facilities across Bamyan and Badakhshan, which are remote provinces in Afghanistan with limited infrastructure and harsh terrain that restrict access to care. To strengthen monitoring and accountability within these facilities, Aga Khan Health Services, Afghanistan, in collaboration with the Aga Khan University, developed and implemented the Sehatmandi mHealth app, a tablet-based tool designed to digitize performance monitoring (Figure 1). The app captures data across 11 key performance indicators, including outpatient visits, antenatal care coverage, institutional deliveries, immunization, and family planning uptake. Its features include monthly target setting, automated dashboard-based visualization, offline data entry for remote sites, and feedback mechanisms for provincial health officers (Figure 2). By enabling real-time or near-real-time reporting, the digital app aims to enhance performance monitoring, workforce productivity, logistical coordination, and accountability across the health system [11].

The Sehatmandi digital app has the potential to support the Afghan Ministry of Public Health’s pay-for-performance framework by enabling data-driven decision-making and strengthening service delivery. With further scale-up, it could contribute to more equitable and sustainable health service provision in Afghanistan [12].

Despite significant efforts, Afghanistan's war-torn health system faces ongoing challenges, largely due to limited health financing [13]. The COVID-19 pandemic further exacerbated the situation by disrupting primary care, highlighting the urgent need for timely, accessible, and relevant information at all levels of the health system to support evidence-based decision-making [14]. Since the Taliban's takeover in August 2021, many donor-funded projects, including the Sehatmandi program, have been halted [15]. Although some health services continue, they now rely on temporary, emergency funding [16]. Therefore, it is crucial to evaluate these interventions to understand their effectiveness in the Afghan context and see if they can be replicated.

Similarly, evaluating the sustainability of digital health tools such as Sehatmandi is vital for understanding their effectiveness and potential for scalability in fragile and conflict-affected regions. Therefore, this study examines the enablers, challenges, and lessons learned from the implementation of Sehatmandi in Afghanistan. By providing evidence of the program's effectiveness, this research aims to help develop strategies and policies for the viability of such interventions in similar settings, ultimately contributing to better health outcomes in LMICs.

## Methods

### Study Design

This study used a qualitative research design, involving in-depth interviews with key health system stakeholders to explore barriers to the sustainability of the Sehatmandi app. The qualitative design allows a rich, nuanced understanding of participant experiences and perspectives.

### Study Setting

The Sehatmandi app was implemented across 189 health facilities, encompassing 115 health facilities in 28 districts of the Badakhshan province and 74 health facilities in 8 districts of the Bamyan province, Afghanistan. For the qualitative study, we selected health facilities across both provinces, ensuring geographic diversity and representation of various facility types. This selection ensured the inclusion of facilities representing varied operational contexts and challenges.

### Study Populations

The study population included health facility managers, administrators, and high-level decision-makers.

#### Inclusion Criteria

Adults (18+ years) who were users of Sehatmandi across catchment facilities in Afghanistan were included.

#### Exclusion Criteria

Users younger than 18 years and those not living in catchment areas were excluded from this study.

#### Recruitment of Participants

A total of 24 participants (14 from Badakhshan and 10 from Bamyan) were recruited using stratified purposive sampling to ensure representation across different facility types and geographic locations. The sampling frame was derived from a structured facility listing that included province, district, facility name, and facility type (eg, Basic Health Center [BHC], Comprehensive Health Center [CHC], Subhealth Center [SHC], stakeholder contact information). Facilities were stratified by province and type, and within each stratum, stakeholders were purposively selected based on their roles and relevance to the Sehatmandi intervention (eg, facility managers, Health Management Information System [HMIS] officers, health administrators). Potential participants were initially contacted by telephone and informed about the study objectives, procedures, and their rights as voluntary participants. Those who agreed were scheduled for interviews and provided written informed consent prior to participation. Recruitment continued until theoretical saturation was reached, defined as the point at which no new themes were emerging from the interviews.

### Data Collection

Semistructured interviews were conducted between June and July 2024 either in a private room at a health facility or virtually via Zoom in cases where physical access was constrained. The interviews were conducted by 2 local qualitative researchers, one from Badakhshan and one from Bamyan, who were familiar with Afghanistan’s sociocultural and health system contexts, enabling strong rapport-building and culturally sensitive engagement. A pretested interview guide comprising open-ended questions was used to elicit in-depth information on participants' experiences with the Sehatmandi app. Interviews were conducted in either Dari or English according to the participant's preference and were audio-recorded with their consent. Transcripts were produced in Dari and subsequently translated into English by bilingual researchers. All translated transcripts were independently verified for accuracy by a third reviewer fluent in both languages. Data collection was concluded upon reaching theoretical saturation, with no new themes emerging, resulting in a total of 24 complete interviews.

### Data Analysis

Thematic analysis was conducted using a deductive-inductive approach. Data were coded and analyzed using NVivo (version 11; QSR International) by a trained researcher at Aga Khan University (FJ). To enhance credibility and interpretive rigor, the research team engaged in reflexive practices such as iterative review of emerging codes, and findings were validated through peer debriefings and team discussions. A realist epistemological stance underpinned the analysis, assuming that participants’ accounts reflect real-world experiences that can inform and sustain digital health interventions in resource-constrained and conflict-affected settings.

### Ethical Considerations

Ethics approval was obtained from the ethical review committee of Aga Khan University, Pakistan (2023-8159-24046) and the institutional review board of the Ministry of Public Health, Afghanistan (A-8-24-444). Informed consent was obtained from all participants prior to the interviews. Participants were informed about the study purpose, voluntary nature of participation, confidentiality, and their right to withdraw at any time without consequence. To ensure privacy and confidentiality, during transcription, all identifiable information was removed from the transcripts. Data were securely stored in password-protected systems accessible only to the research team. No financial compensation was provided.

## Results

### Participants and Themes Identified

A total of 24 participants were interviewed across Badakhshan and Bamyan provinces to evaluate the sustainability of the Sehatmandi digital health intervention in Afghanistan. In Badakhshan, 14 participants were interviewed, comprising 10 health managers, 1 HMIS officer, 1 project director, 1 technical officer, and 1 clinic director. In Bamyan, 10 participants were interviewed, comprising 8 directors of health facilities, 1 HMIS officer, and 1 monitoring and evaluation officer.

Thematic analysis of the qualitative interviews revealed 7 major themes: (1) enhanced performance monitoring and accountability, (2) timely reporting, (3) infrastructure and operational challenges, (4) workforce capacity and training needs, (5) financial and political constraints, (6) monitoring and training gaps, and (7) technical and system limitations.

### Enablers

Participants in this study highlighted key factors that supported the implementation and uptake of the Sehatmandi digital health app. Their perspectives reflect real-world experiences from the field, illustrating how Sehatmandi contributed to improved health system performance. The following themes emerged from the interviews, capturing the ways in which the app enhanced performance monitoring and accountability of health care staff.

One manager explained that the system’s offline functionality enabled data entry in areas with limited internet access.

…Data was entered offline in Sehatmandi and synchronized when the network is available.

Manager, BHC Layaaba, Badakhshan

The platform also streamlined the reporting process to higher administrative levels.

…Reports were sent accurately, correctly, and quickly to the central office.

Manager, Health Facility, Badakhshan

Several participants emphasized how the app supported routine tracking of service delivery metrics, enabling them to monitor progress against key performance indicators. One facility manager reflected as follows.

…Our weekly, daily, and monthly performance was accurately recorded, and we were aware of all our activities. When we collected and recorded data in Sehatmandi, our performance was evident.

Manager, Atin Jalo Health Facility, Badakhshan

The app further facilitated structured review processes during HMIS committee meetings, which helped institutionalize accountability mechanisms.

…The app was acceptable to us because we could keep the clinic staff active. We analyzed the monthly report in the HMIS committee meeting. If any indicator did not meet the target, we would identify the reasons for not reaching the target and instruct the relevant personnel to make more efforts to meet the target in the following month.

Director, Comprehensive Health Center, Shaidan, Bamyan

At the provincial level, a health information officer highlighted the value of Sehatmandi for centralized oversight.

…The Sehatmandi app was well-accepted as a monitoring and reporting tool, providing a simple and effective way to track and oversee health workers' activities.

HMIS Officer, Provincial Office, Bamyan

### Challenges and Lessons Learned

The system faced substantial obstacles, including infrastructure limitations, financial constraints, and human resource challenges, which impeded its overall efficiency and ability to scale. Despite these difficulties, valuable lessons were learned, providing critical guidance for future digital health initiatives in comparable resource-limited and conflict-affected environments.

### Infrastructure and Operational Hurdles

The Sehatmandi digital health intervention faced significant infrastructure and operational hurdles. Inconsistent electricity and poor internet connectivity severely disrupted data synchronization and hindered real-time data monitoring. A few users reported that improper handling led to device breakage. Delays in providing replacements further disrupted consistent data collection. Crucial operational requirements such as timely SIM card distribution were frequently unmet.

…Weak internet at the health center hinders report submission.

Manager, Kalo BHC, Bamyan

…The broken devices were not quickly repaired and made available. Sometimes, it took up to two months to receive a SIM card, and occasionally, the SIM card would be blocked and not quickly reactivated.

Manager, Umal Health Facility, Badakhshan

To enable real-time data monitoring, reliable internet and stable electricity are essential. As a health facility manager emphasized, “All health centers should have access to the internet.” Additionally, durable devices, backup power solutions, and offline data entry options can mitigate disruptions in remote areas. In response, offline data entry was introduced in Sehatmandi, enabling health care administrators to continue data entry without internet connectivity and synchronize the data when connectivity was restored. Moreover, a user highlighted that dedicated local support structure such as an on-site focal person can provide real-time troubleshooting, enhance user confidence, and improve adoption rates.

### Workforce and Training Gaps

High staff turnover and limited digital literacy among health workers initially reduced the usability of the Sehatmandi system. Many health care workers were unfamiliar with digital tools, leading to hesitancy in adoption.

…User unfamiliarity with the application led to incorrect data entry.

Technical Manager, Badakhshan

…Our main problem is the turnover of employees. When a new employee is hired, it takes a lot of time for them to be trained and become proficient, causing delays in work.

Project Director, Provincial Office, Badakhshan

Continuous training and capacity building should be performed to enhance user proficiency and adoption. These training sessions should focus on practical skills, troubleshooting, and building user confidence through structured programs and regular refreshers, ultimately increasing staff proficiency and reducing resistance to the technology, leading to sustainable and improved data collection practices.

…Continuous training should be provided to staff to build their capacity to use application.

Manager, Atin Jawla Health Facility, Badakhshan

### Political Instability and Financial Constraints

Taliban's takeover of Afghanistan in 2021 created significant financial challenges. Several international funding agencies, citing security concerns and uncertainties in governance, suspended funding, abruptly halting project operations. This financial instability limited resource access, demotivated staff, and threatened the program’s sustainability.

…Financial and budgetary issues are the main obstacles.

Manager, BHC Naheya 3, Badakhshan

To ensure sustainability, efforts shall be made to integrate digital health media into national health budgets and explore alternative funding models, including donor support and public-private partnerships. Securing diversified financial resources reduced the dependency on single funding sources and enabled more consistent improvements to the system.

### Monitoring and Evaluation Gaps

Data reliability suffered from inadequate oversight and delayed evaluations. Supervisory teams provided insufficient follow-up, and quality assurance officers were slow to review submitted reports. A manager from a subhealth center stated, “Proper evaluation...was sometimes lacking. Reports we entered were not reviewed for up to two or three months.”

Robust monitoring and feedback systems are crucial for maintaining data accuracy and enhancing overall program performance. Regular monthly follow-up meetings can enable supervisors to provide timely guidance and address emerging problems, fostering accountability and driving continuous improvement. Consistent operational and technical support is also critical for successful app implementation and expansion. As emphasized by a project director, “continuous follow-up is necessary.” Managers also stressed the importance of timely feedback and regular monitoring, with one reporting, “submitted reports should be checked, timely feedback should be provided, and the program should be monitored and followed up.” Furthermore, a shift to more frequent reporting, as suggested by an HMIS manager on a “daily or weekly basis rather than a monthly” could enhance health worker engagement and activity.

### Technical and System Limitations

The Sehatmandi app’s user adoption was hampered by technical and system limitations. For instance, an HMIS manager (Bamyan) noted, “The system requires revision to align with the Ministry of Public Health's new HMIS package. Specifically, integrating reports such as Health Management Information, Monthly Activity and Achievement, Nutrition, Expanded Program on Immunization, Maternal and Child Health, and Pharmacy reports would transform Sehatmandi into a comprehensive data tracking system.”

A manager from the Eshkashem Health Facility (Badakhshan) mentioned that “data were submitted in an aggregated form,” while another manager from the Naheya Health Facility (Badakhshan) stated, “We had to report both through Sehatmandi and on paper, which increased the workload.”

Although a system’s design may align with the administrative goals, its successful adoption by users hinges on how clearly the project’s objectives are communicated. To ensure user buy-in, it is essential to not only explain the project’s purpose but also clearly outline data formats and workflows during trainings.

## Discussion

### Principal Findings

This study evaluates the sustainability of the Sehatmandi digital health intervention in Afghanistan by analyzing perspectives from 24 key stakeholders across Badakhshan and Bamyan provinces. Sehatmandi significantly improved performance monitoring, accountability, and timely data reporting in health facilities, particularly in underserved areas. However, the system faced considerable challenges, including poor infrastructure, financial and political instability, high staff turnover, and limited digital literacy.

### Comparison to Prior Literature

Digital performance monitoring tools, similar to Sehatmandi, are increasingly being adopted in LMICs and have shown measurable benefits in strengthening accountability and workforce performance. A study evaluating Ethiopia’s electronic community health information system found that digitizing performance monitoring and linking it to incentives significantly boosted personnel motivation and performance [17]. Similarly, Pakistan’s digital immunization tracking app improved user satisfaction, microplanning capacity, and institutional transparency among district managers [18]. In South Africa, replacing paper processes with mHealth-based monitoring improved referral tracking and supervision effectiveness [19]. These examples reinforce that combining digital performance dashboards with structured feedback, incentives, and supervision strengthens workforce accountability and performance in resource-constrained health systems.

The challenges identified in this study, particularly infrastructure limitations and workforce constraints, are consistent with findings from digital health implementations in other LMICs. Prior studies have shown that unreliable electricity, poor internet access, and low levels of digital literacy among health care workers are among the most significant barriers to the effective use of mHealth tools in rural and conflict-affected areas [20,21]. The integration of offline functionality in the Sehatmandi app reflects lessons learned from similar interventions in resource-constrained contexts such as Pakistan where hybrid online–offline systems substantially improved usability in regions with intermittent or weak connectivity [22,23].

Human resource challenges were also prominent in this study, particularly high staff turnover and the need for ongoing training to build and sustain digital skills. These findings are widely echoed in the digital health literature, where workforce capacity issues are closely linked to problems with system adoption, data quality, and long-term program sustainability [20,21]. Structured onboarding processes, periodic refresher training, and regular supportive supervision were identified by participants in this study as important strategies to address these barriers. Evidence from Ethiopia further reinforces this view, demonstrating that building the capacity of health workers and performance monitoring teams, implementing incentive structures for data utilization, involving the community in data validation, and establishing accountability systems for health data are crucial for improving data usage and quality [24].

Financial constraints further exacerbate these challenges. The announcement of the cessation of Sehatmandi funding in mid-August 2021 resulted in a sharp decline in health service utilization, emphasizing the critical need for sustained funding to enhance health service delivery [16]. Insufficient funds impede critical infrastructure upgrades, and delays in payment systems further threaten the program’s sustainability. These findings are consistent with the broader systemic issues in health care financing within LMICs, where limited budgets and reliance on donor funding create significant bottlenecks [25].

### Strengths and Limitations

This study offers a number of strengths. First, by including perspectives from 2 distinct provinces, Badakhshan and Bamyan, the findings are geographically and operationally diverse, enhancing the generalizability to similar conflict-affected and low-resource environments. Second, the use of thematic content analysis allowed for a nuanced understanding of the facilitators and barriers to Sehatmandi's sustainability. Third, the rigorous translation and transcription process ensured that the linguistic barriers did not compromise data integrity.

However, there are a few limitations. First, the cross-sectional design provides a snapshot of stakeholder perspectives at a single point in time. This limits the ability to assess temporal changes or the long-term impact. Future research employing longitudinal or follow-up interviews could help track the evolution of digital health adoption over time. Second, this study relies on self-reported data, which may be subject to recall bias or social desirability bias. To mitigate this, participants were assured confidentiality, and interviews were conducted by trained researchers in participants’ local language to encourage openness.

### Future Directions

The findings in this study point toward several future directions. First, investment in infrastructure, particularly internet access at health facilities, durable digital devices, and backup power sources, is crucial for real-time data use in health decision-making. Second, structured and continuous digital literacy training programs for health care workers are essential to overcome resistance, reduce errors, and ensure consistent data quality. Third, system integration with existing national HMIS platforms is critical to avoid fragmentation and to offer a holistic view of health service delivery. Fourth, exploring diversified funding sources, including public-private partnerships and embedding the intervention within national health budgets, could help sustain digital health systems in volatile political climates. Lastly, embedding ongoing monitoring and feedback loops into digital health implementation will foster accountability, adaptability, and continuous learning.

### Conclusion

Participants reported that the Sehatmandi digital health intervention enhanced health system functions, particularly performance monitoring, accountability, and data-driven decision-making, thereby improving service delivery responsiveness in Afghanistan. However, its long-term sustainability and scalability were hindered by infrastructural, technical, human resource, and systemic integration challenges. Adaptive features such as offline data entry helped sustain its use in connectivity-constrained areas. These findings highlight the need for future digital health initiatives in fragile, resource-limited settings to invest in foundational infrastructure, capacity building, follow-up and monitoring, long-term funding, and seamless interoperability with national health systems. Addressing these gaps will enable digital interventions such as Sehatmandi to play a transformative role in improving health outcomes and strengthening health care resilience in LMICs.

## Abbreviations

BHC: basic health center
HMIS: Health Management Information System
LMICs: low- and middle-income countries
mHealth: mobile health

## Data Availability

The datasets generated or analyzed during this study are available from the corresponding author upon reasonable request.
